# Surgical Oncology Heroes and Legends: Dr. Harold Freeman as Interviewed by Dr. Lisa Newman

**DOI:** 10.1245/s10434-026-19300-1

**Published:** 2026-03-18

**Authors:** Harold P. Freeman, Lisa Newman

**Affiliations:** 1 Columbia University, New York City, NY USA; 2 New York-Presbyterian/Weill Cornell Medical Center and Weill Cornell Medicine, New York City, NY USA

The Surgical Oncology Heroes and Legends series is a video program in which leaders of the field are interviewed by colleagues about their many contributions. In succinct, 30-min conversations, these luminaries discuss with other leaders their experiences and insights about surgical oncology.

This episode presents an interview of Dr. Harold P. Freeman by Dr. Lisa Newman; watch the video at https://youtu.be/g1nTEJ5qRSs

**About Dr. Harold P. Freeman**

**Fig. 1:**
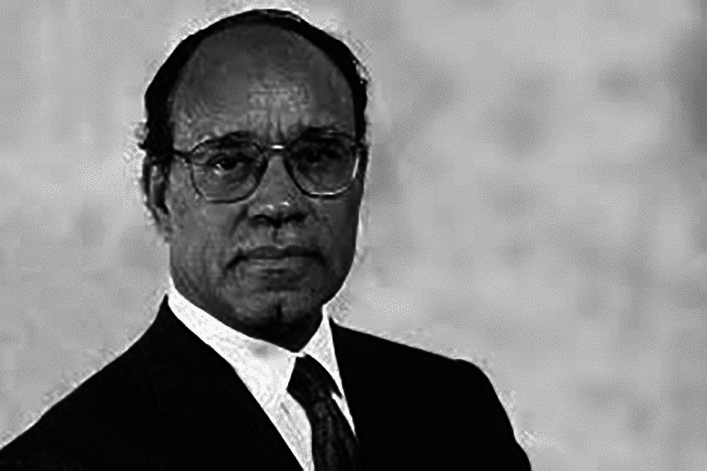
Dr. Harold P. Freeman

Dr. Harold P. Freeman (Fig. 1) is known as the pioneer of the patient navigation concept and is the founder of the Harold P. Freeman Patient Navigation Institute, founder and Chairman Emeritus of the Ralph Lauren Center for Cancer Care and Prevention, Professor of Surgery Emeritus at Columbia University, Attending Surgeon Emeritus at Memorial Sloan Kettering Cancer Center (MSK), and former Director of Surgery at Harlem Hospital, New York, NY. Dr. Freeman began his journey as a cancer surgeon who trained at MSK.

In 1989, Dr Freeman served as the National President of the American Cancer Society (ACS). Prior to that, as a member of the ACS board of directors, Dr. Freeman chaired the subcommittees on Cancer in Minority Americans and later the ACS subcommittee on Cancer in the Socioeconomically Disadvantaged, which led to a report in 1986. This report concluded that Black-White differences in cancer outcomes were primarily due to higher poverty rates in Black Americans.

Related to these conclusions, as President of ACS in 1989, Dr. Freeman led and conducted the ACS National Hearings on Cancer in the Poor, which resulted in the “ACS Report to the Nation on Cancer in the Poor.” The principal finding from these hearings was that poor people of any race face significant barriers when seeking cancer care.

The data acquired from all these studies supported Dr. Freeman’s groundbreaking patient navigation concept and model and led to the passage of the Patient Navigation Act by the US Congress in 2005. In 2024, the Centers for Medicare and Medicaid Services issued a rule that pays for patient navigation services nationwide.

In 2000, Dr. Freeman received the Lasker Award, which he characterizes as the most significant honor of his career. In making this award, the Lasker Foundation stated that Dr. Freeman’s life work has been to address the injustices of bias and discrimination against minorities, especially common in health care, and to ensure that all people receive the best possible care, regardless of their color or income. “Uncommon zeal in the pursuit of his goal, devotion to patients, and a diplomatic, sensitive approach to change characterize his advocacy. Dr. Freeman exemplifies the highest qualities of a public servant on behalf of the underserved.”

He served as chairman of the United States President’s Cancer Panel under the Bush and Clinton administrations. His involvement led to the establishment of the Patient Navigator Outreach and Chronic Disease Act, passed unanimously by the House and Senate at the time. He later served as associate director of the NCI and founding director of the NCI Center for Reducing Cancer Health Disparities. He became a founding president and medical director of the Ralph Lauren Center for Cancer Care and Prevention in Harlem, an MSK site, which provides cancer care and screening for Harlem and surrounding neighborhood residents. In 2007, he established the Harold P. Freeman Patient Navigation Institute and continues to be involved in furthering the patient navigation field for our patients.

**About Dr. Lisa Newman**

Dr. Lisa Newman is an internationally renowned breast surgeon and researcher who serves as Chief of the Section of Breast Surgery at New York-Presbyterian/Weill Cornell Medical Center and Professor of Surgery at Weill Cornell Medicine, New York, NY. At the New York-Presbyterian David H. Koch Center, Dr. Newman leads a multidisciplinary breast oncology program dedicated to providing individualized, cutting-edge care. She also oversees the Breast Surgical Oncology Programs at New York-Presbyterian Lower Manhattan Hospital, Brooklyn Methodist Hospital, and Queens.

As founding medical director of the International Center for the Study of Breast Cancer Subtypes—now headquartered at Weill Cornell Medicine and New York-Presbyterian—Dr. Newman directs an international breast cancer research and training program spanning Ghana, Ethiopia, Nigeria, Uganda, and the Caribbean. She was elected to the National Academy of Medicine in 2023; she serves on the Scientific Advisory Board for the Susan G. Komen Breast Cancer Foundation; and is a Deputy Editor for *Annals of Surgical Oncology*.

**Brief Bibliographies: Top Articles by Dr. Harold P. Freeman**

Dr. Freeman was invited to identify the most important articles of which he is the sole author or a co-author. He provided the following citations. ^1–8^
